# Predictors and consequences of school mobility in middle childhood

**DOI:** 10.1016/j.appdev.2021.101309

**Published:** 2021-08-08

**Authors:** Deborah Lowe Vandell, Megan Kuhfeld, Elizabeth T. Gershoff, Robert Crosnoe

**Affiliations:** a University of California at Irvine, 401 East Peltason, Irvine, CA 92697, United States of America; b NWEA, Portland, OR, United States of America; c Department of Human Development and Family Sciences and the Population Research Center, University of Texas at Austin, 2315 Red River Street, Austin, TX 78712, United States of America; d Department of Sociology and Population Research Center, University of Texas at Austin, 2315 Red River Street, Austin, TX 78712, United States of America

**Keywords:** Peer relationships, Behavior problems, School mobility, Academic achievement, Middle childhood

## Abstract

In this prospective longitudinal study (*N* = 1094, *M* age = 5.6 years to *M* age = 11.1 years), we examined family factors associated with school mobility and then asked if either a move during the previous year or cumulative moves across elementary school were related to child functioning. Family factors were not linked to a recent move or a single move, but changes in family income and household structure did predict higher odds of two or more moves in elementary school. There was no evidence that a recent move or a single move was related to children's academic or social functioning. Effects of two or more moves on child functioning were not significant after controlling for the number of analyses that were conducted. Taken together, school mobility during elementary school did not appear to be a pervasive risk although we were unable to study very high rates of school mobility because of very small sample sizes.

Concern about the negative effects on children of moving from one school to another has been an enduring topic of interest for developmental psychologists, school personnel, and families (Burkam, Lee, & Dwyer, 2009; Coley & Kull, 2016; Dupéré, Archambault, Leventhal, Dion, & Anderson, 2015; Haveman, Wolfe, & Spaulding, 1991; Mehana & Reynolds, 2004). Although school mobility sometimes occurs for positive reasons such as parents' efforts to improve educational opportunities for their children and improvements in family income and circumstances (Hanushek, Kain, & Rivkin, 2004; Anderson, 2017; Rumberger, 2003), research has typically conceptualized school mobility as stressful and disruptive for children. The current study extends the study of school mobility by first identifying family factors that are linked to school mobility and then studying the effects of a school move during the previous year as well as the effects of cumulative moves on children's academic and social functioning during middle childhood.

Scholars have argued that studying school mobility during middle childhood (i.e., elementary school years) is needed for several reasons (Anderson, 2017; Burkam et al., 2009; Schacter, 2004). School mobility is higher in elementary school than in middle school and high school, making it an experience that many children face (Schacter, 2004). Developmentally, changing schools during this period may be challenging for children because this is a time when they are establishing academic footholds in literacy and mathematics that are foundational skills for later achievement (Anderson, 2017; Duncan et al., 2007). Because middle childhood also is an important time for building friendships and peer networks (Buhs, Ladd, & Herald, 2006), social relationships might suffer as a consequence of changing schools. Alternatively, children may take these school moves in stride because they routinely experience new classrooms each year of elementary school, with different teachers and different sets of classmates.

Drawing strong conclusions about the effects of school mobility are complicated by several factors. As underscored by ecological systems theory (Bronfenbrenner & Morris, 2006), school moves are embedded in the broader ecological context of the family. As such, disruptions attributed to school mobility may reflect other aspects of children's lives that co-occur with school moves, rather than the moves themselves (Alexander, Entwisle, & Dauber, 1996; Burkham et al., 2009). In a large study conducted in Tulsa, Oklahoma, for example, school movers were more economically disadvantaged and they less commonly lived in homes with married parents (Anderson, 2017). In the current study, we examine multiple factors that may increase or decrease the odds of school mobility, including children's gender and ethnicity and family factors such as maternal education and psychological adjustment, and family income, and two-parent household structure as well as changes in family income and household structure during middle childhood. We then control for these factors in our examination of the effects of school mobility on children's adjustment in middle childhood.

A second complicating factor related to understanding the effects of school mobility is developmental timing. As highlighted by life course theory (Elder Jr. & Shanahan, 2006), *when* events occur in children's life course is important. Even within middle childhood, the effects of school mobility may vary during the early elementary grades (Kindergarten – 3rd grade) and the later elementary grades (4th – 5th grades) because the academic demands placed on children increase as children progress through school. The primary grades typically place a greater emphasis on teacher-led academic instruction in reading and math whereas the upper elementary grades have more project-based learning with groups of children working together (Pianta, Belsky, Houts, & Morrison, 2007). In the current study, we consider possible effects of developmental timing by examining effects separately for the primary grades and upper elementary school. As noted by Anderson and Leventhal (2017), looking carefully at the timing of when children are changing schools may help determine if more targeted or specialized approaches are needed to meet the needs of children who experience school mobility.

Other aspects of timing also may be important. Both ecological systems theory and life-course theory have emphasized aspects of time such as recent experiences and cumulative experiences because they present different challenges for children. In terms of a recent move, students might initially respond negatively, but then adapt to the social and academic demands of the new school. In this case, there may be evidence of a temporary disruption in children's functioning that quickly dissipates. Alternatively, even a single move could initiate a chain of negative experiences that persists over time. Both ecological systems theory and life course theory lead to predictions that multiple school moves should be more impactful on children's adjustment and learning than a single move because both theories emphasize larger impacts when experiences accumulate over time. Some evidence of potential cumulative effects was reported in a large longitudinal study in the UK that found frequent preschool and elementary school moves between ages 4 and 6 was associated with lower odds of passing achievement assessments at age 6 (Hutchings et al., 2013).

A final issue informed by both ecological systems theory and life course theory is that effects of life experiences such as school mobility can be intensified or ameliorated by family circumstances. That is, the significance of processes in microsystems such as the school or classroom may be dependent on or conditioned by what is occurring in other microsystems, such as the home (Anderson, Leventhal, & Dupéré, 2014; Gasper, DeLuca, & Estacion, 2010). There is some evidence that the stresses associated with school mobility are greater for low-income children (Burkam, et al., 2009). Other circumstances in the family might help to protect children from stresses associated with changing schools. For example, parents' supportive presence for their children's autonomous actions is related to children's academic achievement and emotional well-being during middle childhood (Bradley & Corwyn, 2007; Collins, Madsen, & Susman-Stillman, 2002; NICHD Early Child Care Research Network, 2008). In the current study, we asked if mothers' supportive presence during middle childhood served a protective function for children experiencing school mobility.

Guided by these theoretical perspectives and prior research, the current study examines relations between school mobility and child functioning to determine if there is a general disruption associated with school mobility, or if disruptions are isolated to specific circumstances, or if there is little evidence of significant effects. In addressing these issues, we focus on three domains of development that are important in middle childhood: academic achievement, relationships with peers, and problem behaviors.

## Generalized or specific developmental implications of school moves

Much of the literature examining relations between school mobility and developmental disturbances has been criticized for important limitations. Early studies included limited controls for factors such as family income and changes in family structure that co-vary with school mobility and may account for obtained effects on child functioning (Anderson, 2017; Gasper et al., 2010; Hanushek et al., 2004; Mehana & Reynolds, 2004). Another limitation is that much of the early research used cross-sectional designs to examine concurrent associations between school mobility and developmental outcomes. These designs could not differentiate between short-term and persistent developmental problems or consider processes of cumulative disadvantage (Mehana & Reynolds, 2004). The interactive nature of school moves and other ecological factors such as family income and the quality of mother-child interactions also have not been examined sufficiently, which is important from the vantage point of identifying protective or exacerbating factors that might blunt or reinforce disruption of school moves over time. Finally, comparisons of the implications of school moves for both academic and social-emotional well-being have been limited but needed to determine the degree to which school moves are truly and widely disruptive.

Several longitudinal studies that were motivated by bioecological theory and life course theory have attempted to address these limitations. Using the ECLS-K, Coley and Kull (2016) considered cumulative, timing-specific, and interactive effects of school mobility on cognitive skills, social skills, and internalizing and externalizing behaviors. They found that school mobility was weakly associated with psychosocial functioning, significantly predicting internalizing problems such as sadness and anxiety but not predicting social skills or externalizing behaviors. However, the statistically significant connections between school mobility and internalizing problems were very small across all the models, with effect sizes ranging from only −0.01 to 0.04, raising questions of whether they were meaningful differences. Moreover, due to the gaps in the data collection waves and the method in which school mobility data was measured in the ECLS-K, Coley and Kull were not able to study the cumulative frequency or timing of moves during early elementary school or from kindergarten through 5th grade, which are the focus of the current study.

A second study (Gruman, Harachi, Abbott, Catalano, & Fleming, 2008) used data from the Raising Healthy Children project, a prospective longitudinal study (*n* = 938) conducted at 10 elementary schools in the U.S. Pacific Northwest. Gruman et al. (2008) related school mobility during elementary school to academic performance as reported by teachers and classroom engagement as reported by students. After adding covariates, school changes during the preceding year predicted declines in academic performance and classroom participation but were not related to peer acceptance or teacher support. This study did not examine cumulative moves.

A third study (Dupéré et al., 2015) reported analyses from two prospective longitudinal investigations: the Quebec Longitudinal Study of Child Development conducted in Canada and the NICHD Study of Early Child Care and Youth Development (SECCYD) conducted in the United States. In the Quebec study, information about school moves was collected in 1st, 2nd, 4th, and 6th grades when mothers indicated if their children had changed schools in the previous 12 months and teachers reported the study children's withdrawal, victimization, and aggression. Changing schools in the previous year were unrelated to children having problematic relationships with their peers, with one exception. Teachers reported that children who had experienced a school move *and* a family transition during the previous year were more withdrawn, suggesting that recent moves had short-term effects on child emotional well-being when coupled with a change in family structure.

In their analyses of the SECCYD, Dupéré et al. examined links between school moves and children's peer relationships in 4th and 6th grades, again with special attention to the potential moderating role of a family transition. They found no associations between a school move during the previous year and teacher reports of the study children's relations with peers in 4th and 6th grades. However, there was a suggestion (using one-tailed statistical tests) that the combination of a school move and a family transition predicted the study children's best friend having social problems related to victimization and aggression. School mobility in the absence of a family transition was not associated with teachers' reports of peer-related problems for either the study child or the best friend.

Taken together, these studies involving four large datasets provide countervailing evidence to the notion of generalized pervasive disruptive effects of school mobility on children's academic and social functioning during elementary school. They point, instead, to the need to consider specific time periods, specific developmental domains, and specific family circumstances in which some children may show some negative effects from moving schools. At the same time, the modest number of significant effects (given the number of analyses that were conducted) merit caution in giving undue weight to findings that need replication.

## Study aims

The current study uses data from the NICHD SECCYD to build on and extend previous research by asking if links between school mobility and child development are generalized or specific. Using reports that were regularly obtained from mothers from kindergarten through 5th grade, we identified the exact timing of school moves based on parent-provided dates to address four issues: (1) if school moves during elementary school were predicted by child and family characteristics; (2) if a recent school move (i.e., a move in the previous year) was associated with child developmental outcomes; (3) if cumulative moves were associated with child developmental outcomes; and, finally, (4) if effects associated with school mobility were moderated by family income or sensitive-responsive parenting. We asked if school mobility was related to child functioning during the primary grades when the study children were in 3rd grade and during upper elementary school when the study children were in 5th grade and approaching early adolescence.

Based on prior research, we focused on children's functioning in three domains: peer relationships, problem behaviors, and academic achievement. For peer relationships, we examined children's reports of feeling victimized and feeling lonely. For behavior problems, we examined teacher reports of children's internalizing and externalizing problems. For academic achievement, we used standardized assessments of reading and mathematics achievement. Drawing on prior research and theory, we expected that school mobility would have larger effects on academic achievement in early elementary school when children are building core academic competencies in reading and math. We expected larger effects on peer relationships and problem behaviors in later elementary school when friendships and social peer networks might be more affected by moves. We also expected that cumulative moves would be more disruptive than a single move or a recent move.

## Method

### Participants

SECCYD families were recruited via hospital visits with mothers shortly after the birth of the study children in 1991 in 10 locations in the United States (Little Rock, AR; Irvine, CA; Lawrence, KS; Boston, MA; Hickory, NC; Philadelphia, PA; Pittsburgh, PA; Charlottesville, VA; Seattle, WA; Madison, WI). During selected 24-h intervals, all women giving birth (*N* = 8986) were screened for eligibility. Of those families, 3142 were excluded owing to a priori criteria such as the mother did not speak English, the birth was a multiple birth, and the family planned to move within the next 3 years. At a follow-up telephone interview two weeks later, 1353 families could not be contacted or refused to participate. Among the remaining pool of eligible participants, families were randomly selected for a sample of 1363 families. These families then completed a home interview at 1 month and became the study participants.

In terms of demographic characteristics, 26% of the mothers in the recruited sample had no more than a high school education at the time of enrollment; 21% had incomes no greater than 200% of the poverty level at sixth grade; and 22% were minority (i.e., not non-Hispanic European American). Compared to all births in the United States in 1991, White, non-Hispanic children are somewhat over-represented, and mean household income and maternal education were higher than the U.S. average. For a full discussion of the SECCYD sampling design, see NICHD Early Child Care Research Network (2005).

The data used in this study were approved Study of Early Child Care & Youth Development Phases I – III (IRB HS#2006–5347 at the University of California, Irvine. The data set is publicly available to qualified investigators at https://www.icpsr.umich.edu/icpsrweb/ICPSR/studies/21942.

The analysis sample for the current study consisted of 1094 children. Children were included in the school mobility analyses if they had at least one outcome measure collected between 3rd and 5th grades. To account for missing data, we used the full information maximum likelihood (FIML) procedure in Mplus (Enders, 2001).

## Measures

### School mobility

During the fall and spring of each school year from kindergarten through 5th grade, a parent or other adult guardian, typically the mother, reported information about the schools attended by the study child in the previous year, including start and stop dates, the child's grade level, whether the school was public or private, and the name of the school. NCES school codes were then determined by the data collection sites for each public school that the study children attended, and these codes were used to obtain NCES Common Core Data at the school and district levels for school years 1996–97 through 2005–06.

Based on the school identifiers and start and stop dates, we were able to identify students who moved schools during a school year as well as students who changed schools during the summer. These summer moves could include children moving because they reached the highest grade level of the school (e.g., a school that ends at 2nd grade) as well as children who changed schools because of residential moves or for other reasons.

In order to determine if *recent school moves* were important, we identified moves that had occurred within the past 12 months. Because our measures of child functioning were collected in the spring, a recent school move was defined as one that occurred during the 12 months prior to the spring of the grade of interest. We considered each spring semester to begin in January, thus we focused on moves in the calendar year prior to the spring of the assessment. For example, for 3rd grade outcomes, children were considered to have had a recent move if they changed schools anytime between January and December of the previous year, which corresponded to the spring of 2nd grade, or the summer prior to 3rd grade, or the fall semester of 3rd grade.

For each child, the maternal reports also were used to create a count of *cumulative moves*, which was the total number of school moves from fall of kindergarten through the spring of 2nd grade, as well as the total number of school moves from fall of kindergarten through the spring of 4th grade. By 3rd grade, 733 students had never moved, 260 had moved schools once, and 101 had moved two or more times. By 5th grade, 585 students had never moved, 297 had moved once, and 212 students had moved two or more times. Very frequent moves were rare: only one student had changed schools five times by 3rd grade, and no student had moved more than five times. By 5th grade, 11 students had moved five or more times, and only one student had moved seven times, the threshold identified by the National Research Council and the Institute of Medicine (2010) to designate very frequent movers. Because very frequent movers were so uncommon among the 1094 students, we did not conduct separate analyses for this group.

### Perceived victimization

At 3rd and 5th grades, children reported the extent to which they had been victimized by their classmates (e.g., “Does anyone in your class pick on you at school?”). The average of the four victimization items have been used in several prior studies of children's adjustment to school (Kochenderfer & Ladd, 1996) and are reliable (Cronbach α = 0.81 and 0.85 in 3rd and 5th grades, respectively).

### Loneliness

Children's perceptions of loneliness were assessed during the 3rd and 5th grade home visits using the 24-item Loneliness and Social Dissatisfaction Questionnaire (Asher, Hymel, & Renshaw, 1984). Sixteen items focus on children's feelings of loneliness (e.g., “I feel alone”) and social inadequacy (e.g., “I feel left out of things”), while the remaining eight items are fillers that focus on hobbies or preferred activities. Child loneliness is calculated as the sum of the 16 items (Cronbach α = 0.87 and 0.91 in 3rd and 5th grades, respectively).

### Internalizing behavior problems

Internalizing behavior problems at 3rd and 5th grades were assessed using the age-appropriate version of the Teacher Report Form (TRF) from the Child Behavior Checklist (Achenbach, 1991). The Internalizing scale is based upon the Withdrawn, Somatic Complaints, and Anxious/Depressed subscale items. Raw scores were totaled and converted into age-standardized T scores. The reliability of the internalizing scale (Cronbach α = 0.87 and 0.86 in 3rd and 5th grade, respectively) was high.

### Externalizing behavior problems

Externalizing behavior problems at 3rd and 5th grades were assessed using the age-appropriate version of the Teacher Report Form (TRF) from the Child Behavior Checklist (Achenbach, 1991). The Externalizing scale is based upon the Delinquent and Aggressive subscale items. Raw scores were totaled and converted into age-standardized T scores. The reliability of the externalizing scale (Cronbach α = 0.95 in both 3rd and 5th grade) was high.

### Reading and math achievement

Students' achievement in reading and math were measured using the Woodcock Johnson–Revised (WJ-R) Psycho-Educational Battery (Woodcock & Johnson, 1989). Study children were individually administered the WJ-R subtests by trained research staff in the spring of 3rd grade and 5th grade. The Applied Problems subtest has been widely used to measure math achievement and had good reliability in our sample in both 3rd and 5th grades (Cronbach α = 0.81 and 0.82, respectively). The Letter-Word Identification subtest measures alphabet knowledge and reading achievement. This subtest also demonstrated high internal consistency in 3rd grade (α = 0.90) and 5th grade (α = 0.88).

### Covariates

Several aspects of child, mother, and family characteristics were included as controls for possible selection and omitted variable bias. Basic demographic characteristics were included in all models: the study child's race and ethnicity (White, Black, Hispanic, Other), maternal education in years, the proportion of observation periods during early childhood (6, 15, 24, 36, and 54 months) in which the mother reported that a husband or partner was present, and the average of the family's income-to-needs during these periods. The average of mothers' self-reported depressive symptoms in early childhood assessed with the Center for Epidemiological Studies Depression Scale at 6, 15, 24, 36, and 54 months (α = 0.88–0.91; *r* = 0.46–0.58), was also included in all models. Two measures of early childhood parenting quality were included: an average of standardized ratings of observed maternal sensitivity and observed home environmental quality from the HOME scale (Bradley & Caldwell, 1979) at 6, 15, 24, 36, and 54 months. (See NICHD Early Child Care Research Network (1999, 2003) for a full description of these measures.) Maternal vocabulary, as measured by the Peabody Picture Vocabulary Test–Revised (PPVT–R, Dunn & Dunn, 1981) at the 36-month time point, was included, as was mother's personality measured when the study child was 6 months of age using three subscales (Neuroticism, Extraversion, and Agreeableness, α = 0.91, 0.89, and 0.69, respectively) of the NEO Personality Inventory (Costa & McCrae, 1985). The Early Infant Temperament Questionnaire (Medoff-Cooper, Carey, & McDevitt, 1993) was included to reflect child temperament at 6 months (α = 0.81). The ten sites of the SECCYD were dummy coded (with the largest site used as a referent) and included as covariates.

Two middle childhood aggregate covariates were created to reflect family context in elementary school. The middle childhood income-to-needs aggregate used in the 3rd grade outcome models was the average of income-to-needs reported at 1st and 3rd grades. The middle childhood income-to-needs aggregate used in the 5th grade outcome models was the average of income-to-needs reported at 1st, 3rd, and 5th grades. Maternal supportive parenting was observed and rated during laboratory visits conducted when children were in 1st, 3rd, and 5th grades (see NICHD ECCRN, 2008 for a detailed description of this observational assessment). Middle childhood maternal supportive parenting was the average of the scores reported in 1st and 3rd grades for the 3rd grade outcomes and the average of reported scores in 1st, 3rd, and 5th grades for the analyses of 5th grade outcomes.

Finally, a set of time-varying family covariates was included at each wave to account for family instability during elementary school. Based on mother-reported household rosters, we counted the number of changes in whether a husband or partner was present in the home from kindergarten to the grade of interest. The family income-to-needs ratio change was calculated as the difference between income-to-needs ratio in the grade of interest and their ratio in kindergarten, such that positive values reflect an increase in income-to-needs and negative values reflect a decrease.

## Results

### Descriptive statistics

Table 1 shows descriptive statistics for the key variables in our analyses. Frequent moves were relatively uncommon in early elementary school, with 67% of children not moving in early elementary school (from fall of kindergarten to the summer after 2nd grade), 24% moving once, and 9% moving two or more times. Between kindergarten and the spring of 4th grade, 53% of children had still never moved from one school to another, 27% had moved once, and 19% had moved two or more times.

### Do child and family factors predict school mobility?

We first asked if child and family characteristics predicted school mobility during elementary school. For this question, we used sets of logistic regressions to predict whether students moved in the year prior to 3rd grade or then in the year prior to 5th grade based on child and family characteristics. To ask if child and family characteristics predicted cumulative school moves, we used an ordered logit, or proportional odds, model to first predict if children experienced no school moves, one move, or two or more moves in early elementary school (K-2nd grade) or across elementary school (K-4th grade). Unlike ordinary least squares (OLS) regression, these models do not assume that the outcome variable is ordered on equal intervals. Additionally, ordered logit models are more parsimonious than fitting a series of logistic regressions for each comparison (e.g., no moves vs. one move, one move vs. two or more moves, no moves vs. two or more moves). To develop a single model, ordered logit models estimate cumulative probabilities, namely the probability that the outcome is less than or equal to a given category of the dependent variable. This model estimates regression parameters (*β*) for each independent variable *i* as well as *m* − 1 threshold parameters (*θ*_m_). The ordered logit models reported here assumed that the coefficients that describe the relationship between the odds of moving two or more times versus moving never or once were the same as those that describe the odds of moving one or more times versus never moving. The ordered logit models incorporated the full set of early and middle childhood child and family covariates described above.

Table 2 presents the odds ratios and 95% confidence intervals for the models predicting both recent and cumulative school mobility. The models that examined the odds of moving in the prior 12 months revealed that very few background variables were associated with *recent* moves. Partner-in-home instability and fewer years of mother's education were associated with higher odds of moving in the year before 3rd grade assessment, but no background covariates were associated with moving in the year before the 5th grade assessment.

A change in income-to-needs, partner-in-home instability, and mothers' depressive symptoms in early childhood were each associated with higher odds of cumulative moves before 3rd grade as well as higher odds of cumulative moves before 5th grade. Having a more highly educated mother was associated with moderately lower odds of moving in early elementary school (kindergarten – 2nd grade) but was not associated with the likelihood of moving schools from kindergarten to 4th grade. Higher HOME scores were associated with a lower likelihood of school mobility by both 3rd and 5th grades.

### Are recent school moves linked to children's social adjustment or academic achievement?

Next, a set of OLS models assessed whether *recent school moves*, defined as a move occurring during the 12 months prior to the spring grade of interest, were associated with children's social or academic functioning. Effects associated with having a recent school move were tested in spring of 3rd and in the spring of 5th grade, controlling for the full set of child and family covariates presented in Table 3 (3rd grade) and Table 4 (5th grade). The same six child developmental outcomes were examined in 3rd grade and in 5th grade. To reduce the possibility of Type I errors due to multiple comparisons, we applied the Holm-Bonferroni method to all of the OLS regression results to control the family-wise error rate.

No significant differences were found between children who had experienced a move in the year prior to 3rd grade and children who had not, controlling for child and family background characteristics. In 5th grade, students who had a move in the previous 12 months reported feeling more lonely (B = 0.07, SE = 0.04, *p* < .05) but the difference was not statistically significant after correcting for multiple comparisons. Students who had a recent move in 5th grade did not differ on the other five measures of child functioning from students who did not move.

### Are cumulative school moves related to child developmental outcomes?

Next, we asked if moving more frequently in elementary school predicted children's peer, behavioral, or academic outcomes in 3rd or 5th grade. The first set of OLS models tested cumulative effects of school mobility using indicators for the number of school moves from kindergarten through 2nd grade to predict child functioning at the spring of 3rd grade. For models predicting functioning in 3rd grade, we incorporated all of the child and family covariates collected in early childhood as well as change indicators for each time-varying covariate coded to capture any measured change between the fall of kindergarten and spring of 3rd grade. For models predicting children's functioning in 5th grade, we incorporated all of the child and family covariates collected in early childhood as well as change indicators for each time-varying covariate coded to capture any measured change between the fall of kindergarten and the spring of 5th grade. As with the prior analyses, we applied the Holm-Bonferroni method to all of the OLS regression results to control the family-wise error rate.

Table 5 provides regression results for variables from the fully controlled models for 3rd grade and Table 6 presents regression results for 5th grade. Moving two or more times since kindergarten predicted higher levels of externalizing behavior problems in 3rd grade (B = 0.26, SE = 0.11, *p* < .05). At the end of 5th grade, moving two or more times from kindergarten significantly predicted higher levels of student-reported peer victimization (B = 0.21, SE = 0.09, *p* < .05). However, after we controlled for multiple comparisons, cumulative moves were no longer significantly related to any measures of child functioning in 3rd or 5th grade.

### Are relations between school mobility and child functioning moderated by family factors?

The final sets of analyses re-estimated the regression models adding interaction terms to test whether income-to-needs and maternal sensitivity during middle childhood moderated the associations between school mobility and child functioning in 3rd or 5th grade. These results are shown in Table 7. After accounting for multiple comparisons, there were not any significant interactions between school mobility and either family income or parenting quality.

## Discussion

The current study utilized a large prospective longitudinal sample to ask if school mobility, conceptualized as a recent move during the previous year or as cumulative moves over several years, was linked to child developmental outcomes during middle childhood. In addressing this question, we first identified family factors that co-occurred with school mobility. Then, controlling for family factors, we asked if school moves were associated with children's peer relationships, problem behaviors, and/or academic achievement. The developmental timing of moves was examined by considering school moves in relation to child functioning at two points during middle childhood – first at the end of 3rd grade (early elementary school) and then at the end of 5th grade (late elementary school). Finally, two potential moderators of school mobility – family income and supportive parenting – were tested to determine if effects associated with school mobility were intensified or mitigated by these family factors.

Family factors did not predict if children experienced a *recent move*, suggesting that these moves were idiosyncratic and not consistently related to other factors in the home. Family factors also were not associated with a single move during elementary school. In contrast, two or more cumulative moves were predicted by family factors. Consistent with prior research (Anderson, 2017; Burkham et al., 2009; National Research Council and Institute of Medicine, 2010), we found that changes in family income and changes in family structure (being a two-parent versus single-parent household) increased the odds that children experienced more cumulative moves during early elementary school and across all of elementary school. In addition, lower home quality scores in early childhood and less maternal education increased the odds that children experienced two or more school moves. An implication of these findings is that students who experience higher levels of school mobility are more likely to be also experiencing instability at home and fewer academic and social resources. School personnel including teachers, school psychologists, and counselors need to be sensitive to the needs of students who have experienced several moves. They and their families may benefit from additional instrumental and emotional supports. The absence of consistent associations between family circumstances and recent school moves, in contrast, suggest that children who have experienced a single move or who have recently changed schools do not, as a group, differ from their classmates.

Next, we asked if a recent school move was disruptive for students' social and/or academic functioning. After controlling for the number of comparisons tested, we found no significant associations between recent school moves and six measures of children's academic and social functioning. This lack of robust and consistent associations in middle childhood is similar to other large-scale studies in which family factors were controlled (Coley & Kull, 2016; Dupéré et al., 2016; Gruman et al., 2008), providing some confidence in the observation that children's academic and social functioning is not typically affected by a school move during the preceding year. One reason that recent school moves were not disruptive in these studies may be the result of the organization of schools in the U.S. and Canadian contexts where these studies were conducted. In these countries, children are usually assigned to new classrooms at the beginning of each school year, so having a new teacher and a new group of classmates is an experience shared by all children in the classroom, not just those who changed schools.

Next, we turned to cumulative moves where we contrasted effects associated with a single move versus two or more moves. Here, consistent with the absence of effects of a recent move, we found no evidence that a single move in the year prior to 3rd grade or a single move in the year prior to 5th grade was associated with children's social or academic functioning in any of the models that were tested. We did find some evidence that frequent moves, defined as two or more moves, during early elementary school or across elementary school were related to child outcomes. However, these differences were not statistically significant after we corrected for multiple comparisons. Finally, we asked if effects associated with school mobility were moderated by family factors (family income and/or parenting quality). Among the 12 models that we tested, after controlling for multiple comparisons, we found no evidence that effects of recent moves or cumulative moves varied by family income or supportive parenting.

These findings (or more accurately, lack of findings) indicated that neither a single move nor two or more moves during elementary school was disruptive for children within this large geographically diverse sample. In our view, the general pattern observed in the current study suggests school mobility, as typically experienced by many children in the U.S. context, is not associated with children's social adjustment or academic achievement. As schools prioritize their resources to ensure that their most needy students receive the services they need, school personnel do not need to be generally concerned about students who have experienced a single school move or a recent school move.

At the same time, we are cautious about prematurely assuming that there are *no* risks associated with school mobility, especially if children are experiencing very high levels of mobility or family challenges. Although the SECCYD is geographically and economically diverse, it is not nationally representative, and it contains fewer low-income families than the U.S. as a whole. In addition, we are limited in our ability to evaluate the effects of high levels of school mobility because so few children experienced four, five, or six moves. Indeed, only one student in the current study had moved seven times during elementary school, the threshold identified by the National Research Council and Institute of Medicine Committee (2010) as placing students at high risk. Fortunately, seven or more moves also was very rare in the Early Childhood Longitudinal Study (*N* = 19,000), the nationally representative sample used by Coley and Kull (2016) to study school mobility. For this reason, research that utilizes specifically recruited samples, is needed to illuminate the experiences and developmental outcomes of highly mobile students (National Research Council and Institute of Medicine, 2010). Children who are experiencing high rates of school and residential mobility, including homelessness, may require special services although some of these students perform well at school (Obradovic et al., 2009). When schools and community services adopt a differentiated view of school mobility that incorporates the frequency and timing of school moves in combination with family circumstances, they will be better prepared to serve their students.

A strength of the current study is the detailed records of school mobility, which were collected over a six-year period. Twice each year, mothers reported the names of the schools that the study child had attended. From these reports, we determined which children experienced no school moves between kindergarten and 5th grade, who experienced one move, and who experienced two or more moves. Another strength was that extensive measures of family demographic factors and parenting were used as covariates, and we asked if effects associated with school mobility were moderated by parenting or income mobility may have unique effects.

This study was initially motivated by persistent concerns expressed in the academic and practitioner literature that school moves are stressful for students and negatively impact social and academic functioning. However, the current study, like other recent longitudinal studies, found no generalized or widespread developmental disruptiveness of school mobility during middle childhood. Rather, the evidence suggests that effects of school moves are more constrained with the number of significant effects approaching chance. Thus, although targeted supports are likely needed for highly mobile students – a group not observed in the SECCYD, our results indicate that researchers and practitioners do not need to address normative levels of school mobility and can instead focus their attention on other aspects of children's school experiences that are likely to support the development of elementary school students.

## Figures and Tables

**Table 1:** Descriptive statistics for key independent and dependent variables by wave of the dependent variable.

	3rd Grade (*M* age = 9.02 years)				5th Grade (*M* age = 11.03 years)			
		
Variable	*n*	Wave	M/%	*SD*	*n*	Wave	M/%	*SD*
School mobility								
Recent move (12 months prior)	873	G2-G3	0.17	0.38	841	G4-G5	0.2	0.40
No school move	1094	K-G2	0.67	0.47	1058	K-G4	0.53	0.50
One school move	1094	K-G2	0.24	0.43	1058	K-G4	0.27	0.44
Two or more school moves	1094	K-G2	0.09	0.29	1058	K-G4	0.19	0.40
Outcomes								
Victimization by peers	994	G3	1.84	0.79	976	G5	1.79	0.77
Loneliness	1031	G3	28.47	9.60	1013	G5	25.65	8.98
Internalizing behavior problems	982	G3	51.5	9.60	917	G5	50.45	9.48
Externalizing behavior problems	982	G3	51.51	9.36	917	G5	50.87	9.09
Reading achievement	1014	G3	109.91	14.72	982	G5	108.49	14.50
Math achievement	1013	G3	115.05	15.00	982	G5	109.41	13.53
Child covariates								
Male	1094	1 m	0.51	0.50	1058	1 m	0.51	0.50
Race/ethnicity								
Black	1094	1 m	0.11	0.32	1058	1 m	0.11	0.32
Hispanic	1094	1 m	0.05	0.22	1058	1 m	0.05	0.22
Other	1094	1 m	0.06	0.23	1058	1 m	0.06	0.23
Early childhood covariates								
Mother's education (in years)	1094	1 m	14.43	2.46	1058	1 m	14.43	2.46
Mother's PPVT score	1026	36 m	99.85	18.22	996	36 m	99.85	18.22
Mother's psychological adjustment	1063	6 m	59.19	13.75	1031	6 m	59.19	13.75
Mother has partner in home	1094	1-54 m	0.85	0.31	1058	1-54 m	0.85	0.31
Mother's depressive symptoms	1094	1-54 m	9.73	6.51	1058	1-54 m	9.73	6.51
Income-to-needs ratio	1086	1-54 m	3.71	2.84	1052	1-54 m	3.71	2.84
Average HOME score	1087	1-54 m	40.2	4.65	1053	1-54 m	40.2	4.65
Mother's sensitive parenting	1048	1-54 m	17.14	2.46	1018	1-54 m	17.14	2.46
Child's temperament	1070	6 m	3.17	0.40	1037	6 m	3.17	0.40
Mother's work hours	1094	1-54 m	18.73	13.38	1058	1-54 m	18.73	13.38
Middle childhood covariates								
Mother's supportive parenting	1064	K-G3	16.6	2.46	1026	K-G5	16.54	2.21
Income-to-needs ratio	1054	K-G3	4.19	3.39	1021	K-G5	4.34	3.53
Time-varying covariates								
Income-to-needs change	902	K-G3	0.85	2.11	898	K-G5	1.03	2.39
Number of partner-in-home changes	939	K-G3	0.15	0.43	876	K-G5	0.26	0.61

*Note*: M = mean; *SD* = standard deviation; m = months; K = kindergarten; G2 = 2nd grade; G3 = 3rd grade; G4 = 4th grade; G5 = 5th grade; PPVT = Peabody Picture Vocabulary Test; HOME = Home.

Observation for Measurement of the Environment.

**Table 2:** Odds ratios (and 95% Confidence Intervals) for models predicting school mobility.

	School mobility by 3rd grade		School mobility by 5th grade	
		
	Recent move within 12 months	Cumulative moves (K- G2)^a^	Recent move within 12 months	Cumulative moves (K- G4) ^a^
Male	1.29 (0.83,1.99)	1.11 (0.85,1.44)	0.98 (0.68,1.40)	0.96 (0.76,1.23)
Black	1.3 (0.63,2.67)	1.13 (0.69,1.83)	0.88 (0.46,1.66)	1.06 (0.67,1.68)
Hispanic	1.92 (0.82,4.49)	1.36 (0.75,2.45)	0.96 (0.41,2.21)	1.28 (0.72,2.26)
Other	0.73 (0.26,2.06)	0.84 (0.47,1.51)	0.61 (0.24,1.56)	0.85 (0.50,1.45)
Mother's education (in years)	0.82** (0.71,0.93)	0.92* (0.85,0.99)	0.96 (0.86,1.07)	0.94 (0.88,1.01)
Mother's PPVT score	1.01 (1.00,1.03)	1.01 (0.99,1.02)	1 (0.99,1.02)	1 (0.99,1.01)
Mother's psychological Adjustment	1.01 (0.99,1.03)	1 (0.99,1.02)	1 (0.98,1.02)	1 (0.99,1.01)
Mother has partner in home (EC)	0.57 (0.28,1.19)	0.95 (0.58,1.54)	0.7 (0.37,1.32)	0.83 (0.52,1.33)
Mother's depressive symptoms (EC)	1.03 (1.00,1.08)	1.03* (1.00,1.05)	1 (0.96,1.03)	1.02 (1.00,1.05)
Income-to-needs ratio (EC)	0.95 (0.78,1.15)	1.01 (0.91,1.12)	1.03 (0.88,1.20)	0.97 (0.88,1.06)
Average HOME score (EC)	0.95 (0.89,1.02)	0.94** (0.91,0.99)	0.99 (0.94,1.05)	0.95* (0.92,0.99)
Mother's sensitivity (EC)	1.1 (0.98,1.23)	1.02 (0.95,1.09)	1.03 (0.94,1.14)	1.04 (0.98,1.12)
Child's temperament (6 months)	0.98 (0.54,1.75)	1 (0.71,1.41)	1.01 (0.64,1.61)	0.97 (0.71,1.34)
Mother's work hours (EC)	0.98 (0.97,1.00)	0.99 (0.98,1.00)	0.99 (0.98,1.01)	0.99 (0.98,1.00)
Income-to-needs change (MC)	0.97 (0.80,1.17)	1.17** (1.05,1.29)	0.94 (0.82,1.08)	1.08* (1.00,1.17)
Number of partner-in-home changes (MC)	1.64* (1.04,2.56)	1.42* (1.03,1.96)	1.2 (0.89,1.61)	1.32* (1.06,1.66)
Mother's supportive parenting (MC)	0.96 (0.85,1.07)	0.99 (0.92,1.07)	0.96 (0.86,1.06)	0.94 (0.87,1.01)
Income-to-needs aggregate (MC)	1.06 (0.89,1.28)	0.94 (0.85,1.04)	0.96 (0.83,1.11)	0.96 (0.88,1.04)

*Note:* K = kindergarten; G2 = 2nd grade; G4 = 4th grade; PPVT = Peabody Picture Vocabulary Test; EC = early childhood, i.e., the average of the variable reported at 1-, 6-, 15-, 24-, 36-, and 54-month interviews; HOME = Home Observation for Measurement of the Environment; MC = middle childhood, i.e., the average of the variable reported at 1st and 3rd grade for the third grade models and average of the variable reported at 1st, 3rd, and 5th grades for the fifth grade models. The comparison group for Black, Hispanic and Other race or ethnicity was White.

a Cumulative moves is an ordinal variable with three categories: no moves, one move, or two or moves.

* *p* < .05.

** *p* < .01.

**Table 3:** OLS regression estimates of the associations between recent school moves and child functioning in 3rd grade.

	Perceived victimization	Loneliness	Internalizing behavior	Externalizing behavior	Reading achievement	Math achievement
						
	B (SE)	B (SE)	B (SE)	B (SE)	B (SE)	B (SE)
Recent move	0.02 (0.04)	0.04 (0.03)	0.01 (0.04)	0.05 (0.03)	0.03 (0.03)	0.05 (0.03)
Male	−0.07 (0.06)	−0.03 (0.06)	−0.04 (0.06)	−0.02 (0.06)	−0.04 (0.06)	0.08 (0.06)
Black	0.26 (0.12)*	0.28 (0.12)*	−0.16 (0.12)	0.22 (0.12)	−0.26 (0.12)*	−0.40 (0.11)***
Hispanic	0.08 (0.15)	0.07 (0.15)	−0.05 (0.15)	−0.22 (0.14)	0.04 (0.14)	0.08 (0.14)
Other	0.06 (0.14)	0.08 (0.14)	0.05 (0.15)	−0.09 (0.14)	0.01 (0.13)	−0.03 (0.13)
Mother's education (in years)	−0.02 (0.02)	−0.02 (0.02)	−0.01 (0.02)	−0.02 (0.02)	0.04 (0.02)*	0.03 (0.02)*
Mother's PPVT score	0.15 (0.04)**	0.05 (0.04)	0.03 (0.05)	0.05 (0.04)	0.19 (0.04)***	0.11 (0.04)**
Mother's personality score	−0.04 (0.04)	0.00 (0.04)	0.05 (0.04)	−0.05 (0.04)	−0.07 (0.04)	−0.09 (0.04)*
Mother has partner in home (EC)	0.10 (0.13)	0.09 (0.13)	−0.18 (0.13)	−0.28 (0.13)*	0.01 (0.12)	−0.02 (0.12)
Mother's depressive symptoms (EC)	0.01 (0.01)	0.00 (0.01)	0.01 (0.01)	0.00 (0.01)	0.00 (0.01)	−0.01 (0.01)
Income-to-needs ratio (EC)	−0.08 (0.06)	−0.08 (0.05)	0.01 (0.06)	0.08 (0.05)	−0.03 (0.06)	−0.00 (0.05)
Average HOME score (EC)	0.01 (0.05)	−0.07 (0.05)	−0.04 (0.05)	−0.14 (0.05)**	0.15 (0.04)**	0.20 (0.04)***
Mother's sensitivity (EC)	−0.12 (0.04)**	−0.02 (0.04)	−0.10 (0.04)*	−0.13 (0.04)**	0.03 (0.04)	0.08 (0.04)*
Child's temperament (6 months)	−0.04 (0.03)	0.01 (0.03)	−0.03 (0.03)	−0.01 (0.03)	−0.04 (0.03)	−0.02 (0.03)
Mother's work hours (EC)	0.00 (0.00)	0.00 (0.00)	−0.00 (0.00)	0.00 (0.00)	0.00 (0.00)	0.00 (0.00)
Income-to-needs change (K-G3)	−0.03 (0.03)	−0.04 (0.03)	−0.08 (0.04)*	−0.06 (0.03)	−0.03 (0.05)	0.04 (0.03)
Number of partner-in-home changes (K-G3)	−0.11 (0.16)	−0.05 (0.15)	0.23 (0.16)	−0.10 (0.16)	0.07 (0.03)*	−0.03 (0.14)
Mother supportive parenting (K-G3)	−0.03 (0.04)	−0.05 (0.04)	−0.07 (0.04)	−0.12 (0.04)**	0.06 (0.04)	0.05 (0.04)
Average income-to-needs ratio (K-G3)	0.10 (0.17)	0.02 (0.17)	−0.26 (0.18)	0.09 (0.17)	0.09 (0.08)	0.07 (0.16)

*Note. N* = 1094. Standard errors are in parentheses. PPVT = Peabody Picture Vocabulary Test; EC = early childhood, i.e., the average of the variable reported at 1-, 6-, 15-, 24-, 36-, and 54-month interviews; HOME = Home Observation for Measurement of the Environment; K = kindergarten; G3 = 3rd grade. Models also controlled for site. The comparison group for Black, Hispanic and Other race or ethnicity was White. Bold values indicate significant values after correcting for multiple comparisons.

* *p* < .05.

** *p* < .01.

*** *p* < .001.

**Table 4:** OLS regression estimates of the associations between recent school moves and child functioning in 5th grade.

	Perceived victimization	Loneliness	Internalizing behavior	Externalizing behavior	Reading achievement	Math achievement
						
	B (SE)	B (SE)	B (SE)	B (SE)	B (SE)	B (SE)
Recent move	−0.00 (0.04)	0.07 (0.04)*	−0.02 (0.04)	−0.01 (0.04)	0.04 (0.03)	−0.01 (0.03)
Male	0.02 (0.06)	0.12 (0.06)	−0.04 (0.06)	0.04 (0.06)	0.03 (0.06)	0.12 (0.06)*
Black	0.18 (0.12)	−0.07 (0.12)	0.02 (0.13)	0.47 (0.13)***	−0.20 (0.11)	−0.45 (0.11)***
Hispanic	−0.08 (0.15)	0.06 (0.14)	0.00 (0.15)	−0.10 (0.15)	0.24 (0.14)	0.32 (0.14)*
Other	−0.06 (0.14)	−0.02 (0.14)	0.10 (0.14)	0.19 (0.14)	0.13 (0.13)	−0.03 (0.13)
Mother's education (in years)	−0.02 (0.02)	0.01 (0.02)	−0.03 (0.02)	−0.00 (0.02)	0.04 (0.02)*	0.04 (0.02)*
Mother's PPVT score	0.14 (0.04)**	0.07 (0.04)	0.05 (0.05)	0.01 (0.05)	0.19 (0.04)***	0.13 (0.04)**
Mother's personality score	−0.06 (0.04)	0.01 (0.04)	−0.05 (0.04)	−0.04 (0.04)	−0.06 (0.04)	−0.06 (0.04)
Mother has partner in home (EC)	0.16 (0.13)	0.01 (0.13)	0.16 (0.14)	−0.06 (0.13)	−0.07 (0.11)	0.13 (0.12)
Mother's depressive symptoms (EC)	0.01 (0.01)*	0.02 (0.01)**	0.00 (0.01)	−0.01 (0.01)	0.00 (0.01)	−0.01 (0.01)
Income-to-needs ratio (EC)	−0.06 (0.06)	−0.09 (0.06)	−0.07 (0.06)	−0.01 (0.06)	−0.07 (0.06)	−0.01 (0.05)
Average HOME score (EC)	−0.03 (0.05)	−0.03 (0.05)	−0.06 (0.05)	−0.15 (0.05)**	0.19 (0.04)***	0.13 (0.04)**
Mother's sensitivity (EC)	−0.04 (0.04)	−0.05 (0.04)	−0.03 (0.04)	−0.04 (0.04)	0.02 (0.04)	0.08 (0.04)*
Child's temperament (6 months)	−0.01 (0.03)	−0.01 (0.03)	0.03 (0.03)	−0.04 (0.03)	−0.05 (0.03)	−0.06 (0.03)*
Mother's work hours (EC)	0.01 (0.00)**	0.00 (0.00)	−0.00 (0.00)	0.00 (0.00)	0.00 (0.00)	0.00 (0.00)
Income-to-needs change (K-G5)	−0.06 (0.03)	−0.04 (0.03)	−0.08 (0.03)*	−0.09 (0.03)**	−0.02 (0.04)	−0.04 (0.03)
Number of partner-in-home changes (K-G5)	−0.09 (0.10)	−0.06 (0.10)	0.05 (0.11)	0.17 (0.11)	0.05 (0.03)	0.10 (0.10)
Mother supportive parenting (K-G5)	−0.07 (0.04)	−0.01 (0.04)	−0.06 (0.04)	−0.12 (0.04)**	0.10 (0.04)*	0.06 (0.04)
Average income-to-needs ratio (K-G5)	0.07 (0.12)	−0.00 (0.12)	−0.04 (0.13)	−0.17 (0.12)	0.12 (0.07)	0.01 (0.11)

*Note. N* = 1094. Standard errors are in parentheses. PPVT = Peabody Picture Vocabulary Test; EC = early childhood, i.e., the average of the variable reported at 1-, 6-, 15-, 24-, 36-, and 54-month interviews; HOME = Home Observation for Measurement of the Environment; K = kindergarten;; G5 = 5th grade. Models also controlled for site. The comparison group for Black, Hispanic and Other race or ethnicity was White. Bold values indicate significant values after correcting for multiple comparisons.

* *p* < .05.

** *p* < .01.

*** *p* < .001.

**Table 5:** OLS regression estimates of the associations between cumulative school mobility and child functioning at 3rd grade.

	Perceived victimization	Loneliness	Internalizing behavior	Externalizing behavior	Reading achievement	Math achievement
						
	B (SE)	B (SE)	B (SE)	B (SE)	B (SE)	B (SE)
One school move (K-G2)	0.05 (0.08)	0.08 (0.07)	0.02 (0.08)	0.07 (0.07)	−0.13 (0.07)	−0.08 (0.07)
Two or more school moves (K-G2)	−0.06 (0.11)	−0.05 (0.11)	0.07 (0.12)	0.26 (0.11)*	0.18 (0.10)	0.12 (0.10)
Male	−0.08 (0.06)	−0.03 (0.06)	−0.04 (0.06)	−0.03 (0.06)	−0.05 (0.06)	0.08 (0.06)
Black	0.26 (0.12)*	0.30 (0.12)*	−0.14 (0.12)	0.23 (0.12)	−0.27 (0.12)*	−0.42 (0.11)***
Hispanic	0.08 (0.15)	0.09 (0.14)	−0.03 (0.15)	−0.22 (0.14)	0.04 (0.14)	0.08 (0.14)
Other	0.07 (0.14)	0.08 (0.14)	0.08 (0.15)	−0.06 (0.14)	0.01 (0.13)	−0.04 (0.13)
Mother's education (in years)	−0.03 (0.02)	−0.02 (0.02)	−0.01 (0.02)	−0.02 (0.02)	0.04 (0.02)**	0.03 (0.02)*
Mother's PPVT score	0.17 (0.04)***	0.05 (0.04)	0.03 (0.05)	0.06 (0.04)	0.19 (0.04)***	0.11 (0.04)**
Mother's personality score	−0.03 (0.04)	0.00 (0.04)	0.05 (0.04)	−0.04 (0.04)	−0.07 (0.04)	−0.09 (0.04)*
Mother has partner in home (EC)	0.03 (0.12)	0.04 (0.12)	−0.24 (0.13)	−0.35 (0.12)**	0.02 (0.12)	0.03 (0.11)
Mother's depressive symptoms (EC)	0.01 (0.01)	0.00 (0.01)	0.01 (0.01)	0.00 (0.01)	0.00 (0.01)	−0.01 (0.01)
Income-to-needs ratio (EC)	−0.07 (0.07)	−0.09 (0.06)	0.03 (0.07)	0.13 (0.06)	−0.02 (0.06)	−0.03 (0.06)
Average HOME score (EC)	−0.02 (0.05)	−0.07 (0.05)	−0.02 (0.05)	−0.15 (0.05)**	0.15 (0.04)**	0.20 (0.04)***
Mother's sensitivity (EC)	−0.13 (0.04)**	−0.01 (0.04)	−0.09 (0.04)*	−0.13 (0.04)**	0.03 (0.04)	0.08 (0.04)*
Child's temperament (6 months)	−0.04 (0.03)	0.01 (0.03)	−0.03 (0.03)	−0.02 (0.03)	−0.04 (0.03)	−0.03 (0.03)
Mother's work hours (EC)	0.00 (0.00)	0.00 (0.00)	−0.00 (0.00)	0.00 (0.00)	0.00 (0.00)	0.00 (0.00)
Income-to-needs change (K-G3)	−0.01 (0.05)	−0.03 (0.05)	0.04 (0.06)	0.04 (0.05)	−0.02 (0.05)	−0.03 (0.05)
Number of partner-in-home changes (K-G3)	−0.11 (0.03)**	0.06 (0.03)	0.07 (0.04)*	0.00 (0.04)	0.06 (0.03)*	−0.02 (0.03)
Mother supportive parenting (K-G3)	−0.03 (0.04)	−0.05 (0.04)	−0.06 (0.04)	−0.12 (0.04)**	0.07 (0.04)	0.04 (0.04)
Average income-to-needs ratio (K-G3)	−0.00 (0.08)	−0.00 (0.08)	−0.08 (0.08)	−0.07 (0.08)	0.08 (0.08)	0.08 (0.07)

*Note. N* = 1094. Standard errors are in parentheses. K = kindergarten; G2 = 2nd grade; G3 = 3rd grade; PPVT = Peabody Picture Vocabulary Test; EC = average of the variable reported at 1-, 6-, 15-, 24-, 36-, and 54-month interviews; HOME = Home Observation for Measurement of the Environment. Models also controlled for site. The comparison group for Black, Hispanic and Other race or ethnicity was White. Bold values indicate significant values after correcting for multiple comparisons.

* *p* < .05.

** *p* < .01.

*** *p* < .001.

**Table 6:** OLS regression estimates of the associations between cumulative school mobility and child functioning at 5th grade.

	Perceived victimization	Loneliness	Internalizing behavior	Externalizing behavior	Reading achievement	Math achievement
						
	B (SE)	B (SE)	B (SE)	B (SE)	B (SE)	B (SE)
One school move (K-G4)	0.01 (0.07)	−0.01 (0.07)	0.05 (0.08)	−0.06 (0.07)	0.00 (0.07)	−0.00 (0.07)
Two or more school moves (K-G4)	0.21 (0.09)*	0.11 (0.09)	0.02 (0.09)	0.15 (0.09)	0.02 (0.08)	0.03 (0.08)
Male	0.02 (0.06)	0.12 (0.06)	−0.04 (0.06)	0.03 (0.06)	0.03 (0.06)	0.11 (0.06)
Black	0.20 (0.12)	−0.06 (0.12)	0.05 (0.13)	0.51 (0.12)***	−0.21 (0.11)	−0.44 (0.11)***
Hispanic	−0.10 (0.15)	0.04 (0.15)	0.01 (0.16)	−0.10 (0.15)	0.24 (0.14)	0.32 (0.14)*
Other	−0.05 (0.14)	−0.02 (0.14)	0.10 (0.14)	0.21 (0.14)	0.12 (0.13)	−0.02 (0.13)
Mother's education (in years)	−0.01 (0.02)	0.01 (0.02)	−0.02 (0.02)	0.00 (0.02)	0.04 (0.02)*	0.04 (0.02)*
Mother's PPVT score	0.14 (0.04)**	0.08 (0.04)	0.05 (0.05)	0.01 (0.05)	0.19 (0.04)***	0.13 (0.04)**
Mother's personality score	−0.06 (0.04)	0.00 (0.04)	−0.05 (0.04)	−0.04 (0.04)	−0.06 (0.04)	−0.06 (0.04)
Mother has partner in home (EC)	0.14 (0.12)	−0.02 (0.13)	0.10 (0.13)	−0.12 (0.13)	−0.08 (0.11)	0.09 (0.12)
Mother's depression (EC)	0.01 (0.01)*	0.02 (0.01)**	0.00 (0.01)	−0.01 (0.01)	0.00 (0.01)	−0.00 (0.01)
Income-to-needs ratio (EC)	−0.08 (0.06)	−0.10 (0.06)	−0.05 (0.07)	0.04 (0.07)	−0.07 (0.06)	−0.00 (0.06)
Average HOME score (EC)	−0.01 (0.05)	−0.02 (0.05)	−0.06 (0.05)	−0.15 (0.05)**	0.19 (0.04)***	0.13 (0.04)**
Mother's sensitivity (EC)	−0.04 (0.04)	−0.05 (0.04)	−0.03 (0.04)	−0.04 (0.04)	0.02 (0.04)	0.08 (0.04)*
Child's temperament (6 months)	−0.01 (0.03)	−0.01 (0.03)	0.03 (0.04)	−0.04 (0.03)	−0.06 (0.03)	−0.06 (0.03)*
Mother's work hours (EC)	0.01 (0.00)**	0.00 (0.00)	−0.00 (0.00)	0.00 (0.00)	0.00 (0.00)	0.00 (0.00)
Income-to-needs change (K-G5)	−0.03 (0.04)	−0.04 (0.04)	0.03 (0.05)	0.09 (0.05)	−0.02 (0.04)	0.01 (0.04)
Number of partner-in-home changes (K-G5)	0.03 (0.04)	0.02 (0.04)	0.04 (0.04)	−0.03 (0.04)	0.05 (0.03)	−0.03 (0.03)
Mother supportive parenting (K-G5)	−0.07 (0.04)	−0.01 (0.04)	−0.06 (0.04)	−0.13 (0.04)**	0.10 (0.04)*	0.06 (0.04)
Average income-to-needs ratio (K-G5)	0.01 (0.07)	−0.03 (0.07)	−0.04 (0.08)	−0.11 (0.08)	0.12 (0.07)	0.09 (0.07)

*Note. N* = 1094. Standard errors are in parentheses. K = kindergarten; G4 = 4th grade; G5 = 5th grade; PPVT = Peabody Picture Vocabulary Test; EC = early childhood, i.e., the average of the variable reported at 1-, 6-, 15-, 24-, 36-, and 54-month interviews; HOME = Home Observation for Measurement of the Environment. Models also controlled for site. The comparison group for Black, Hispanic and Other race or ethnicity was White. Bold values indicate significant values after correcting for multiple comparisons.

* *p* < .05.

** *p* < .01.

*** *p* < .001.

**Table 7:** OLS regression estimates of the associations between cumulative school mobility and child functioning at 5th grade by key moderators.

	Perceived victimization by peers	Loneliness	Internalizing behavior	Externalizing behavior	Reading achievement	Math achievement
						
	B (SE)	B (SE)	B (SE)	B (SE)	B (SE)	B (SE)
Moderator: Average income-to-needs ratio (MC)						
One school move (K-G4)	0.01 (0.07)	−0.00 (0.07)	0.05 (0.08)	−0.06 (0.07)	0.00 (0.07)	−0.00 (0.07)
Two or more school moves (K-G4)	0.22 (0.13)	0.20 (0.12)	0.11 (0.13)	0.17 (0.13)	−0.06 (0.12)	−0.13 (0.12)
Income-to-needs average (K-G5)	0.02 (0.07)	−0.03 (0.07)	−0.03 (0.08)	−0.11 (0.08)	0.11 (0.07)	0.08 (0.07)
Interaction between two or more moves and income-to-needs	−0.00 (0.05)	−0.04 (0.04)	−0.04 (0.05)	−0.01 (0.04)	0.04 (0.04)	0.08 (0.04)
Moderator: Maternal supportive parenting (MC)						
One school move (K-G4)	0.01 (0.07)	−0.00 (0.07)	0.06 (0.07)	−0.06 (0.07)	0.00 (0.07)	−0.00 (0.07)
Two or more school moves (K-G4)	0.66 (0.41)	0.55 (0.37)	0.72 (0.34)*	0.23 (0.43)	−0.26 (0.44)	−0.28 (0.46)
Maternal supportive parenting (K-G5)	−0.05 (0.04)	0.00 (0.04)	−0.04 (0.04)	−0.13 (0.04)*****	0.09 (0.04)*	0.05 (0.04)
Interaction between two or more moves and maternal supportive parenting	−0.18 (0.16)	−0.18 (0.14)	−0.28 (0.13)*	−0.03 (0.17)	0.11 (0.17)	0.12 (0.18)

*Note. N* = 1094. Standard errors are in parentheses. K = kindergarten; G4 = 4th grade; G5 = 5th grade; MC = middle childhood. All predictor variables displayed were standardized. All models presented include the full list of control variables shown in previous models: gender, race or ethnicity, mother's education, mother's Peabody Picture Vocabulary Test score, mother's psychological adjustment at 6-months, Home Observation Measurement of the Environment score averaged across early childhood, mother's sensitivity averaged across early childhood, family income-to-needs ratio averaged across early childhood, mother's supportive parenting averaged across middle childhood, family income-to-needs ratio averaged across middle childhood, average number of maternal work hours across early childhood, partner in home averaged across early childhood, number of partner in home changes from kindergarten to fifth grade, difference in income-to-needs between kindergarten and fifth grade, child's temperament at 6 months, mother's depressive symptoms, and site. No coefficients were significant after correcting for multiple comparisons.

* *p* < .05.

** *p* < .01.

*** *p* < .001.
